# Drowning

**Published:** 1859-08

**Authors:** 


### DROWNING.

The following directions, issued by the National Life Boat Institution of England, should be in the pocket book of every traveller, for the purpose of applying them for the restoration of persons apparently drowned.

1. Treat the patient instantly, on the spot, in the open air—exposing the face and chest to the breeze, except in severe weather.

2. *To clear the Throat*—Place the patient gently, face downwards, with one wrist under the forehead, in which position all fluids will escape by the mouth, and the tongue itself will fall forward, leaving the entrance into the windpipe free. Assist this operation by wiping and cleansing the mouth. If there be breathing—wait and watch; if not, or if it fail, then,

2. *To Excite Respiration*—Turn the patient well and instantly on the side, and

4. Excite the nostrils with snuff, hartshorn, volatile salts, or the throat with a feather, &c., and dash cold water on the face, previously rubbed warm. If there be no success, lose not an instant, but begin

5. *To Imitate Respiration*—Replace the patient on the face, raising and supporting the chest on a folded coat or other article of dress.

6. Turn the body very gently on the side and a little beyond, and then briskly on the face, alternately, repeating these motions deliberately, efficiently and perseveringly, about fifteen times in the minute, or every four seconds, occasionlly varying the side.

7. On each occasion that the body is replaced on the face, make uniform but efficient pressure, with brisk movement on the back between and below the shoulder blades on each side, removing the pressure immediately before turning the body on the side. The result is respiration or natural breathing, and if not too late, life.

8. After respiration has been restored, promote the warmth of the body by the application of hot flannels, bottles or bladders of warm water, heated bricks, &c., to the pit of the stomach, the arm pits, between the thighs and to the soles of the feet.

9. *To Induce Circulation and Warmth.*—During the whole time do not cease to rub the limbs upwards, with firm, grasping pressure, and with energy, using handkerchiefs, flannels, &c.

10. Let the limbs be thus warmed and dried, and then clothed, the bystanders furnishing the requisite garments.

*Cautions.*—1. Send quickly for medical assistance, and dry clothing.

2. Avoid all rough usage and turning the body on the back.
3. Under no circumstances hold up the body by the feet.
4. Nor roll the body on casks.
5. Nor rub the body with salts or spirits.
6. Nor inject tobacco smoke or infusion of tobacco.
7. Avoid the continuous warm bath.
8. Be particularly careful to prevent persons crowding around the body.

*General Observations.*—On the restoration of life a teaspoonful of warm water should be given, and then, if the power of swallowing have returned, small quantities of wine, or brandy and water, or coffee. The patient should be kept in bed, and a disposition to sleep encouraged.

The treatment recommended should be persisted in for a considerable time, as it is an erroneous opinion that persons are irrecoverable because life does not soon make its appearance, cases having been successfuly treated after persevering several hours.

Study carefully the above rules, and lay them by for future reference, and some person may have occasion to thank you for preserving his life by your preserving them.

